# The structural characterization and bioactivity assessment of nonspecific lipid transfer protein 1 (nsLTP1) from caraway (*Carum carvi*) seeds

**DOI:** 10.1186/s12906-023-04083-9

**Published:** 2023-07-20

**Authors:** Taibah Aldakhil, Saud O. Alshammari, Bushra Siraj, Bishoy El-Aarag, Shamshad Zarina, David Salehi, Aftab Ahmed

**Affiliations:** 1 Biomedical and Pharmaceutical Sciences, Chapman University School of Pharmacy, Irvine, CA 92618 USA; 2 Department of Pharmaceutical Chemistry, College of Pharmacy, Prince Sattam Bin Abdulaziz University, Al-Kharj, 16278 Saudi Arabia; 3 Department of Plant Chemistry and Natural Products, Faculty of Pharmacy, Northern Border University, Arar, 91431 Saudi Arabia; 4 Dr. Zafar H. Zaidi Center for Proteomics, University of Karachi, Karachi, Pakistan; 5 Biochemistry Division, Chemistry Department, Faculty of Science, Menoufia University, Shebin El-Koom, 32512 Egypt

**Keywords:** Caraway, Nonspecific lipid transfer protein, Molecular modeling, Phylogenetic tree, Cytotoxicity, Antioxidant

## Abstract

**Background:**

*Carum carvi* (caraway) of the Apiaceae family has been used in many cultures as a cooking spice and part of the folk medicine. Previous reports primarily focus on the medicinal properties of caraway seed essential oil and the whole seeds extract. However, no effort has been made to study caraway proteins and their potential pharmacological properties, including nonspecific lipid transfer protein (nsLTP), necessitating further research. The current study aimed to characterize nonspecific lipid transfer protein 1 (nsLTP1) from caraway seed, determine its three-dimensional structure, and analyze protein–ligand complex interactions through docking studies. We also evaluated nsLTP1 in vitro cytotoxic effect and antioxidant capacity. Additionally, nsLTP1 thermal- and pH- stability were investigated.

**Methods:**

Caraway nsLTP1 was purified using two-dimensional chromatography. The complete amino acid sequence of nsLTP1 was achieved by intact protein sequence for the first 20 residues and the overlapping digested peptides. The three-dimensional structure was predicted using MODELLER. Autodock Vina software was employed for docking fatty acids against caraway nsLTP1. Assessment of nsLTP1 cytotoxic activity was achieved by MTS assay, and the Trolox equivalent antioxidant capacity (TAC) was determined. Thermal and pH stability of the nsLTP1 was examined by circular dichroism (CD) spectroscopy.

**Results:**

Caraway nsLTP1 is composed of 91 residues and weighs 9652 Da. The three-dimensional structure of caraway nsLTP1 sequence was constructed based on searching known structures in the PDB. We chose nsLTP of *Solanum melongena* (PDB ID: 5TVI) as the modeling template with the highest identity among all other homologous proteins. Docking linolenic acid with caraway protein showed a maximum binding score of -3.6 kcal/mol. A preliminary screening of caraway nsLTP1 suppressed the proliferation of human breast cancer cell lines MDA-MB-231 and MCF-7 in a dose‑dependent manner with an IC_50_ value of 52.93 and 44.76 μM, respectively. Also, nsLTP1 (41.4 μM) showed TAC up to 750.4 μM Trolox equivalent. Assessment of nsLTP1 demonstrated high thermal/pH stability.

**Conclusion:**

To the best of our knowledge, this is the first study carried out on nsLTP1 from caraway seeds. We hereby report the sequence of nsLTP1 from caraway seeds and its possible interaction with respective fatty acids using in silico approach. Our data indicated that the protein had anticancer and antioxidant activities and was thermally stable.

**Supplementary Information:**

The online version contains supplementary material available at 10.1186/s12906-023-04083-9.

## Background

Plant rituals are primarily the shared practice amongst traditional health systems, even though every culture has its own practices. Various parts of plants have been frequently used as conventional remedies worldwide. Besides, numerous extracted small molecule phytochemicals, the plant bioactive compounds, continually contribute to drug discovery. According to Newman & Cragg (2020), "Natural products still hold out the best options for finding novel agents/active templates" to produce new lead compounds for drug discovery if combined with emerging techniques [1]. Nonetheless, the plant's biologically active proteins and peptides are underestimated areas in the nutraceutical industry that could promise drug discoveries. Although complementary medicine has been used for different human ailments, the major drawback is the lack of scientific evidence-based practice [2]. This study aimed to explore and contribute to a better understanding of the structure–function relationship of a biologically active protein from this valuable medicinal herb. Further, to answer its pharmacological importance as a prospective anticancer agent employing sensitive cell-based biological assay using breast cancer cell lines, including triple-negative breast cancer cell line.

Nonspecific lipid transfer proteins (nsLTPs) are present in various plant species and expressed extensively in most tissues. The first reported LTP was isolated by Kader (1975) from potato tuber (*Solanum tuberosum*) [3]. Though new classifications emerged, conventional classification is still in use and classifies them based on the molecular mass as 10 kDa for nsLTP1 and 7 kDa for nsLTP2 [4]. NsLTPs are cysteine-rich proteins that contain eight cysteine motifs connected to form four disulfide bridges. These bonds stabilize the protein structure against high temperature and denaturing agents. Moreover, their tunnel-like structure forms an internal hydrophobic cavity that can bind to different lipids and transfer them [5]. Therefore, its name comes from its general role, mediating nonspecific lipids transfer between membranes in the cytoplasm. They also exhibit lipid sensing, lipid presenting, and lipid-modifying functions [6].

Lipid transfer proteins are expressed in various plants' seeds, leaves, stems, roots, flowers, and fruits. Due to the diversity in lipid transfer proteins among plants, the physiological function of nsLTP1 is not completely understood. However, inhibition of LTP gene expression results in a number of hypotheses regarding their possible role in the development of plants during their vegetative and reproductive phases and a decrease in their resistance to infection [5]. Their capacity to bind and transfer lipids is considered accountable for many of their functions, and their abundance would imply their critical roles in plant survival and reproduction. Numerous studies discovered nsLTPs involvement in plant vital roles, e.g., signaling, cuticular wax accumulation, liquid secretion, seed germination, cell expansion, nodule formation, and root suberin synthesis [7]. In addition, nsLTPs are critical in pollen, seed, and fruit development. Their variability and transferability might be promising for drug discovery and drug delivery studies. Various reported nsLTPs exhibited biological activities, for example, antiproliferative activity against MCF-7, AsPC-1, HL-60, and HepG2 human cancer cells; antifungal activity against *C. tropicalis* and *C. albicans*; antibacterial activity against *S. aureus* and *P. aeruginosa*; antiviral activity against RSV, H1N1, and HIV-1 reverse transcriptase; and enzyme inhibition activity against human salivary α-amylase [5, 8–11].

The cultivation and consumption of caraway in Europe are believed to exist longer than any other spice. *Carum carvi* is a plant that belongs to the family Apiaceae. *C. carvi* fruit is dry and indehiscent; therefore, known as a caraway seed [12, 13]. Caraway seed was a common aromatic spice in cuisines and baked goods in many cultures. It is also integrated into their folk medicine for indigestion, flatulence, appetite loss, galactagogue, pneumonia, and eczema [12]. The seeds of caraway are still used in traditional medicine. Many studies have recently investigated caraway seeds' bioactivities through in vitro, in vivo, and clinical studies. In vitro*,* studies showed antimicrobial, antioxidant, anti-diabetic, anti-inflammatory, and chemopreventive effects [14, 15]. Besides, in vivo*,* experiments displayed anti-colitic activity, anti-convulsant, hepatoprotective, and wound healing properties [14–16]. In clinical trials, caraway exhibited an anti-obesity effect, functional dyspepsia management, and irritable bowel syndrome soothing [14, 15]. The terpenes in caraway essential oil, predominantly carvone, and limonene, are the active components responsible for caraway bioactivity [14].

While caraway is commonly used in folk medicine, its scientific studies are limited. To date, the whole caraway seed extract and essential oil bioactivities have been studied, but there is a lack of studies on their proteins. This study focuses on the primary structure characterization of nsLTP1 from the caraway seeds by N-terminal amino acid sequencing. In addition, the three dimensional structural modeling, lipid binding potential, and a phylogenetic tree were achieved using several bioinformatics tools. Furthermore, prospective biological anticancer and antioxidant activity studies have also been performed. The study also evaluated its secondary structure conformation stability at different temperatures and pH levels using circular dichroism (CD) spectroscopy.

## Materials and methods

### Protein extraction

Caraway seeds were morphologically authenticated by Dr. Muneeba Khan, Taxonomist at the Center for Plant Conservation, Karachi University Herbarium, and Botanical Garden, University of Karachi, Pakistan. As per institutional policy, seeds are not accepted as a voucher specimen, and no deposition number is issued required for the whole plant.

Ground caraway seeds immersed in n-hexane for 24 h defatted and dried in a fume hood. Defatted caraway seeds were continually stirred in Phosphate-Buffered Saline (PBS), pH 7.4, for 24 h at 4 °C. Extracted samples were filtered through mesh filter fabric and centrifuged at 14,000 rpm for 30 min. Then, the supernatant was precipitated using 80% ammonium sulfate and stirred for 24 h at 4 °C. Precipitated proteins were recovered by centrifugation at 14,000 rpm for 30 min. The pelleted proteins were dialyzed in water at 4 °C and lyophilized.

### Electrophoresis

Tris/tricine Sodium dodecyl sulfate–polyacrylamide gel electrophoresis (SDS-PAGE) with 10% resolving gel and 4% stacking gel was performed for the crude protein, as well as the collected chromatography fractions during each step of the purification process. The resulting bands were stained by Coomassie blue dye followed by water destain.

### Gel filtration chromatography

Using FPLC AKTA pure (GE Healthcare), the gel filtration column HiLoad 26/600 Superdex 200 pg separated proteins as the first stage of the two-dimensional separation. The dried protein mixture dissolved in PBS was centrifuged, and the supernatant was injected into FPLC. The running buffer was PBS, pH 7.4, at 2.6 ml/min, and the absorbance of the proteins was detected at 280 nm.

### Reversed-phase chromatography

The RP-HPLC was used to purify the proteins using Aeris 3.6 µm WIDEPORE C4 250 × 4.6 mm (Phenomenex). The running solvents consist of water containing 0.1% TFA (solvent A) and acetonitrile containing 0.1% TFA (solvent B). Gradient programming of the solvent system was carried out at 0–60% solvent B for 55 min. The absorbance was monitored at 280 nm. The column Nucleodur C18 (MACHEREY–NAGEL) was used for peptide purification experiments, and the absorbance was adjusted to 214 nm.

### ***MALDI-TOF mass spectrometry***

The matrix 3,5-Dimethoxy-4-hydroxycinnamic acid (SPA) was prepared in 50% acetonitrile containing 0.1% Trifluoroacetic acid (TFA). 1 μl of SPA and 1 μl of nsLTP1 (dissolved in 0.1% TFA) were mixed, spotted on the MALDI plate, and placed in Autoflex MALDI-TOF (Bruker) to determine the protein's molecular weight (m/z).

### Protein modification by 4-vinylpyridine

The purified proteins were dissolved in reduction and alkylation buffer composed of Guanidine/HCl 6 M, tris base 0.2 M, di-sodium EDTA, 2 mM, and 5 μl of 2-mercaptoethanol (βME) in a ratio of 1:1. Under nitrogen blowing, βME was added (1:10) and incubated at 50 ℃ for 4 h. Then, 4-vinylpyridine was added to the solution (1:10) and incubated for 1.5 h at 37 °C. The reaction was quenched by βME containing 5% acetic acid (1:1).

### Enzymatic digestion

The pyridylethylated protein was digested by Trypsin, TPCK-treated (Sigma-Aldrich) to a final enzyme to protein ratio of 1:20 (w/w) in 50 mM Tris/HCl, pH 8.3 for 4 h at RT. Then, the digestion was quenched with 1 M acetic acid to pH 4, and RP-HPLC separated the resulting peptides.

### ***Cyanogen bromide cleavage***

The purified protein (1 mg) was dissolved in 2 ml of 70% formic acid. Then, 4 mg of Cyanogen Bromide (CNBr) was added to the solution at 25 °C and incubated in the dark for 24 h. A vacuum concentrator dried the cleaved methionyl bond peptides to isolate them by RP-HPLC.

### Amino acid sequencing

The N-terminal amino acid sequence of the intact protein and the purified peptides were determined by automatic Edman degradation using a PPSQ-51A/53A gas-phase protein sequencer (Shimadzu). The retention time of the PTH-amino acid residues was compared to a PTH-amino acid standard for identification. Approximately 20 pmol of the protein/peptide of interest was spotted on a PVDF membrane, and the first 30 amino acid sequence was performed.

### Alignment and construction of the phylogenetic tree

As a query sequence, representative nsLTP1 sequences from different genera were obtained from NCBI blastp against caraway nsLTP1. The alignment of multiple sequences retrieved from NCBI was carried out using the Muscle program [17]. The sequence alignment data was analyzed by the Jalview tool. MEGA 11 software [18] was used to perform the phylogenetic analysis. The neighbor-joining method was used to construct the phylogenetic tree. The parameters were set as default according to the selected method, which uses Jones–Thornton–Taylor (JTT) model for pairwise distance matrix. Bootstrapping was performed to calculate the percentage of taxa clusters with a bootstrap value of 1000 (1000 replicates).

### Prediction of protein structure

The homology modeling approach was used to predict the three-dimensional structure of caraway nsLTP1. The amino acid sequence was subjected to blastp [19] against the PDB database (Protein Data Bank) to look for the closest similarity of protein structures. 5TVI was the best template based on identity, similarity, and other statistical parameters. Using 5TVI as the reference structure, the model of the amino acid sequence was built by MODELLER v9.23 [20]. Modeller constructs the structure of a protein target by a comparative modeling approach, which uses the structure of a known protein. The software generated five default models. The best homology model was chosen based on the least negative DOPE score. The model was superimposed on the template structure to see the difference by RMSD calculation through visualizing software UCSF Chimera [21].

### Evaluation of modeled structure

The modeled structure of caraway nsLTP1 protein was analyzed through SAVES v6.0 structure validation server, and the Ramachandran plot was assessed using PROCHECK [22]. The Ramachandran plot was used to examine the stereochemical quality by calculating phi and psi angles of amino acid residues. Additionally, the modeled protein was also evaluated through the ProSA web server [23], which indicates potential structure errors. It calculates the residues' z-score and energy plot, thus considering the model's overall quality.

### Ligand and receptor preparation

Structures of fatty acids, i.e., linolenic, palmitic, and linoleic acid, were obtained from PubChem Database [24] and stearic acid from ChemSpider [25]. Both databases provide a vast collection of chemical molecules for biological assays. All the structures were converted into the required format of the molecular docking tool, prepared by adding hydrogen atoms, and saved in PDBQT format through the AutoDock tool (ADT). Likewise, the modeled protein was prepared in ADT by adding Kollman charges and polar hydrogen for utilization in the docking program. Further, the binding pocket of modeled structure was predicted by the DoGSite Scorer server [26]. This server generates a grid around protein, applies Gaussian filter difference, and identifies favorable locations by making spheres on the grid positions. The potential pocket is selected based on the maximum density threshold of each pocket cluster.

### Molecular docking

Molecular docking is a structure-based approach employed to explore the binding orientation attained by ligands and the interaction pattern between receptor and ligand. In this regard, AutoDock Vina [27] software was run for docking fatty acids against the modeled structure. The grid box was set around the residues of modeled protein defined by the DoGSite server. The x, y, and z dimensions of the grid box were adjusted by 38, 38, and 34, and the box was centered by 86.954, 35.12, and -10.554 x, y, and z coordinates, respectively. As a result, nine default conformers were obtained from the docking of each ligand. Discovery Studio Visualizer [28] was used for the docking analysis. The analysis was carried out considering the binding affinities and non-covalent interactions in a protein–ligand complex, including hydrogen bonding, hydrophobic, and van der Waal interactions.

### Cell culture

Triple-negative breast cancer (MDA-MB-231) and human breast cancer (MCF-7) cell lines were purchased from ATCC (Manassas, USA), cultured, and maintained in DMEM medium supplemented with 10% (v/v) of FBS and 1% penicillin–streptomycin. Cells were incubated at 37 ºC humidified at a 5% CO_2_ incubator.

### MTS assay

The in vitro antiproliferation effect of nsLTP1 against MDA-MB-231 and MCF-7 cells was determined using 3-(4,5-dimethylthiazol-2-yl)-5-(3-carboxymethoxyphenyl)-2-(4-sulfophenyl)-2H-tetrazolium (MTS) assay. In a 96-well plate, 10,000 cells/well were seeded and incubated for 24 h at 37 ºC humidified 5% CO_2_ incubator. Then, a final concentration range (0–95 μM) of caraway nsLTP1 was added, and cells were incubated for 48 h. After that, the media was discarded, and fresh media was added containing 0.5 mg/mL MTS. After 4 h, the absorbance was recorded at 490 nm by a microplate reader. The percentage of cell inhibition was calculated as follows:

$$\%\;\mathrm{Cell\;in\;hibition}=\frac{\begin{array}{c}(\mathrm{Absorbance\;of\;untreated\;cells}-\\\mathrm{absorbance\;of\;treated\;cells})\end{array}}{\mathrm{Absorbance\;of\;untreated\;cells}}\mathrm x100$$

### ***IC***_***50***_*** determination***

Three independent triplicate experiments were used to graph the dose–response curve over a range of concentrations by nonlinear regression analysis using Prism 9.5.0 (GraphPad).

### Total antioxidant capacity assay

The total antioxidant capacity (TAC) assay kit (MAK334, Sigma-Aldrich) was used to detect the antioxidant activity of nsLTP1 according to the manufacturer's instructions. Trolox standards were prepared from Trolox stock (50 mM) by combining 5 μL of standard with 245 μL of ultrapure water. Then, the standards were diluted from (0–1000 μM). The assay was started by transferring 20 μL of standards and caraway nsLTP1 into a flat bottom 96-well plate in triplicate. Then, 100 μL of the reaction mixture (100 μL of reagent A and 8 μL of reagent B) was added to all assay wells, and then the plate was incubated for 10 min at room temperature. The absorbance was measured at 570 nm (*A*_570_) through a microplate reader, and the blank *A*_570_ value was subtracted from standard and sample values. The standard curve was generated by plotting Trolox concentrations against their correspondence *A*_570_. Total antioxidant capacity is expressed as μM equivalent of Trolox, calculated using the following equation.

$$\mathrm{TAC}\;\left(\mathrm{uM}\right)=\frac{\left({\mathrm A}_{570\;}\right)\mathrm{sample}-\left({\mathrm A}_{570}\right)\;\mathrm{blank}}{\mathrm{Slope}\;(\mathrm{uM}^{-1})}\times\mathrm n$$

where: (*A*_570_) *sample* = the absorbance of the sample

(*A*_570_) *blank* = the absorbance of the medium blank.*n* = sample dilution factor.

### CD spectroscopy

Caraway nsLTP1 (15 μM), dissolved in ultrapure water, was used for CD spectroscopy analysis. The following parameters were applied using a J-1500 spectropolarimeter (Jasco) equipped with the Peltier PTC-510 temperature controller (Jasco) under the following conditions: Nitrogen flow 20 SCFH, PM-539 detector, data interval 0.1 nm, data pitch 0.1 nm, CD scale 200 mdeg/0.1 dOD, FL scale 200 mdeg/0.1 dOD, bandwidth 1 nm, cell length 1 mm, start mode immediately, scanning mode continuous, scanning speed 50 nm/min, and shutter control auto. An average of three readings between 190 and 260 nm was collected for each sample. The background of ultrapure water was subtracted. For the temperature-dependent CD spectroscopy, the sample was scanned at 20, 40, 60, 80, and 95 °C after 20 min of heating at the specified temperature. After collecting the data at 95 °C, the sample was kept at 20 °C for 20 min. Then, the sample was scanned again at 20 °C. Under the same conditions and parameters, lysozyme was used as a control to compare the conformational changes in the secondary structure after the thermal treatment. For pH-dependent CD spectroscopy, samples in artificial gastric pH 1.6 and intestinal pH 6.8 fluids were scanned at 37 °C. Analysis was carried out by Spectra Manager software (Jasco) using principal component regression (PCR), with a basis set containing 26 proteins reference set under the following conditions: standardization of result 100, replaced negative value to zero, and rejection percentage 1%.

### Statistical analysis

Prism 9.5.0 (GraphPad) was used to analyze the experimental data. One-way ANOVA was used for group comparison, followed by Tukey’s post-hoc test to determine the statistical significance of the antioxidant assay. Results with *P* values of < 0.05 were considered statistically significant and presented as *P < 0.05, **P < 0.01, ***P < 0.001.

## Results 

### Purification of nsLTP1

**Fig. 1:**
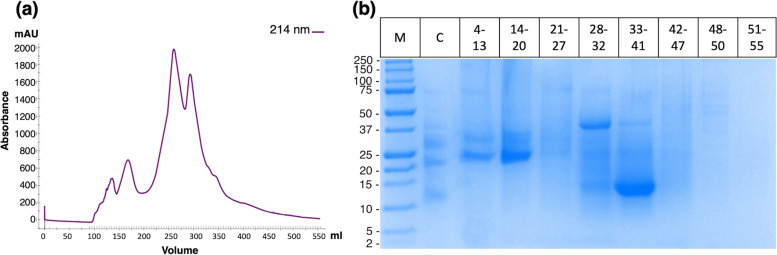
Isolation of caraway proteins. **a** Fractionation profile of proteins from caraway (*Carum carvi*) on HiLoad 26/600 Superdex 200 pg column. **b** Electrophoretic profile by Tris/Tricine SDS-PAGE (10%) of caraway seeds proteins precipitates and gel filtration chromatography fractions. Lane M, standard molecular weight marker; Lane C, crude proteins; and Lane 1–8, eluted gel filtration fractions

**Fig. 2:**
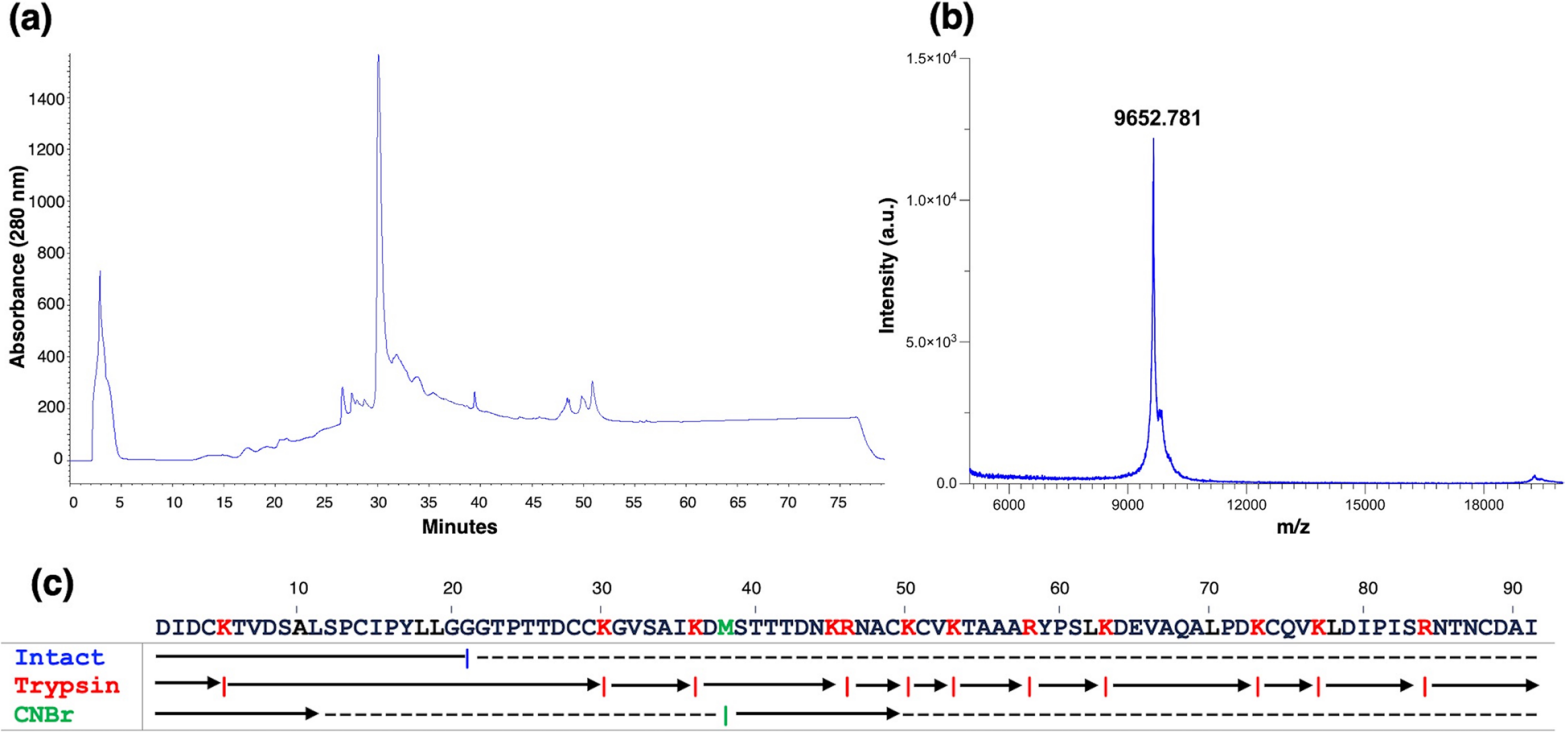
Chromatographic profile and analysis of caraway nsLTP1. **a** 2D-RP-HPLC elution profile of pooled gel filtration fractions containing caraway nsLTP1 on Aeris 3.6 µm WIDEPORE C4 250 × 4.6 mm column. **b** MALDI-TOF mass spectra of RP-HPLC purified caraway nsLTP1. **c** Amino acid sequence of nsLTP from caraway seeds. Solid line represent the N-terminal sequence of intact protein, while arrows represent cleaved peptides sequenced after trypsin and CNBr digestion

The crude proteins were extracted from defatted caraway seeds in PBS pH 7.4 at 4 °C, and protein precipitation was accomplished using 60% ammonium sulfate successfully. Gel filtration chromatography partially separated the protein mixture (Fig. 1a). 10%Tris/Tricine SDS-PAGE gel displayed a protein band around 9–10 kDa in the pooled fractions 33–41 (Fig. 1b), and the full-length gel figure is present in Supplementary Figure S1. The pooled fraction was further purified by RP-HPLC that yielded a highly purified protein eluted at 30 min (Fig. 2a). MALDI-TOF mass spectrometry determined the protein mass at *m/z* 9652 Da (Fig. 2b).

### Amino acid sequence of nsLTP1

The N-terminal Edman sequencing of intact protein identified the amino acid residues up to 20 residues (DIDCKTVDSALSPCIPYLLG). The cysteine residues were successfully modified using 4-vinylpyridine and recognized as PE Cystein in Edman sequencing. The protein was subjected to TPCK-treated trypsin digestion to determine the primary sequence completely. The produced peptides were purified by RP-HPLC and sequenced. Based on the homology with other nsLTPs, the peptide linkage was assigned. The absence of arginine and lysine from one of the peptides is implicit as the C-terminal peptide.

Moreover, employing CNBr cleavage reaction yielded two fragmented peptides that validated the presence of a single Met residue at position 38. When aligned with Trypsin peptides, CNBr peptide-2 revealed the presence of -Lys-Arg- at residues 45–46. Combining the intact protein sequence data, trypsin digestion, and CNBr cleavage, peptides elucidate the complete primary structure of caraway seeds nsLTP1 consisting of 91 amino acids (Fig. 2c). The nsLTP1 contains eight conserved cysteine residues that form and retain its typical tertiary folding. The protein sequence data reported in this paper will appear in the UniProt Knowledgebase under the accession number C0HM61.

### Multiple sequence alignment

**Fig. 3:**
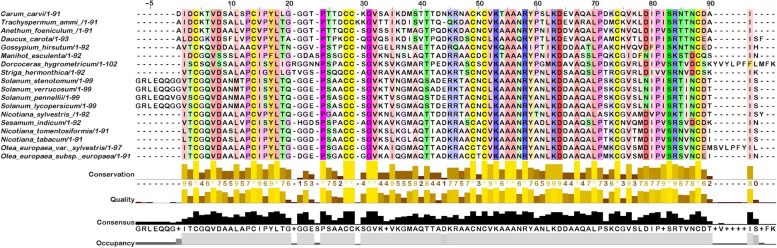
Multiple sequence alignment of caraway nsLTP1 performed through Muscle program. Amino acids in multiple sequences are colored on the basis of conserved residues by Jalview. Scale bar at the top shows the position of amino acids. Left side represents several nsLTPs. The golden bar displays the substitution that occurred due to subtle changes in amino acids. Consensus bar in black color indicates the most common conserved residues of the alignment. *Carum carvi* sequence was set as a reference for numbering convention

Global alignment of multiple nsLTP1 sequences was performed, as shown in Fig. 3. Cysteine is a highly conserved residue in all nsLTP1 homologs. Additionally, Val7, Leu18, Pro24, Gly32, Val33, Lys53, Arg58, Ala67, Leu70, and Ser83 are also conserved in caraway nsLTP1 and all analyzed nsLTP1 sequences. The sequence of caraway nsLTP1 showed a maximum identity of 77.78% with the sequence of ajwain nsLTP1 (*Trachyspermum ammi*) and the least identity of 50.5% with multiple sequences including *Solanum stenotomum*, *Solanum verrucosum*, and *Gossypiuum hirsutum*.

### Phylogenetic analysis

**Fig. 4:**
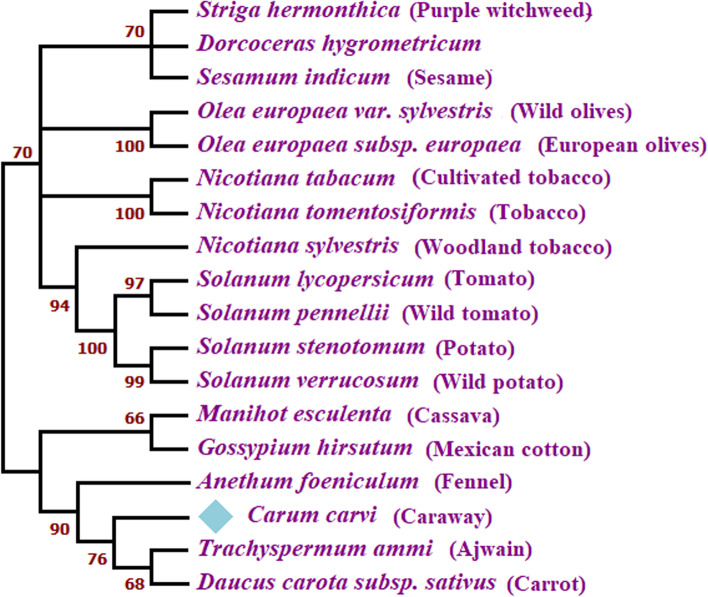
Neighbor-joining based unrooted phylogenetic tree of nsLTP protein sequences by MEGA11 software. Bootstrap value is present at each node. Diamond shape denotes caraway as a reference sequence

An unrooted phylogenetic tree was analyzed by aligning different nsLTP protein sequences using the neighbor-joining method. Homologous sequences were excluded from the result of the blastp homology search for alignment purposes. The nodes of tree were assessed by 1000 repetitions. Figure 4 shows that the tree contains several families of nsLTP sequences. From the top, a clade with bootstrap value 70 is divided into branches as well as its sub-branches consisting of nsLTPs of different taxonomy as well as Solanaceae family members, i.e., *Nicotiana* and *Solanum*. The other side of a clade is joined with *Manihot esculenta*, *Gossypium,* and members of the Apiaceae family. In Apiaceae, *C. carvi* (caraway), *T. ammi* (ajwain)*, Anethum foeniculum* (fennel), and *Daucus carota* (carrot) are clustered together, in which *C. carvi* shares the most common ancestor with *T. ammi* and *D. carota*. Besides, the rest of the tree contains many other branches having species of different genera.

### Hom*ology modeling*

**Fig. 5:**
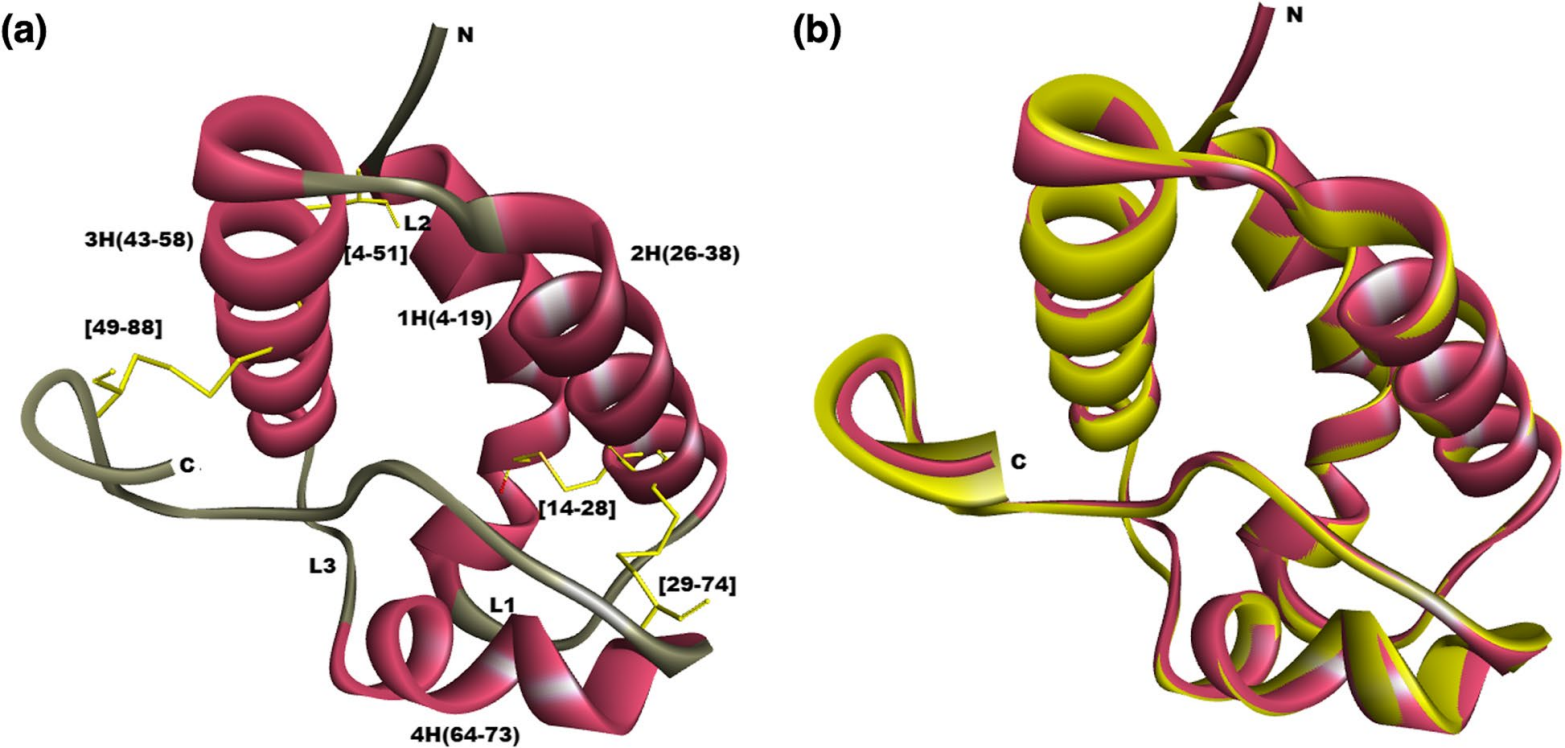
Three-dimensional structure of caraway nsLTP1 in ribbon style. **a** Modeled caraway nsLTP1. N and C represent two terminals (basic and acidic end) of protein. 1H, 2H, 3H, and 4H represent four helices present in this protein model, along with their positions. Cysteine residues making disulfide bridges are highlighted with yellow and loops are colored grey. **b** Superimposed structure of modeled caraway (in pink color) with its template structure 5TVI (in yellow)

The three-dimensional structure of caraway nsLTP1 sequence was constructed with the help of searching known structures available in protein databases. BLAST (Basic Local Alignment Search Tool) was used to input the protein query. We chose nsLTP1 of *Solanum melongena* (PDB ID: 5TVI) as the modeling template of caraway nsLTP1 with the highest identity of 47.78%, similarity 67%, lowest E-value 5e^−29^ and query coverage of 98% among all other homologous proteins (Supplementary Table S1). MODELLER program aligned target and template, i.e., caraway nsLTP1 and 5TVI, and built different models. Among the models obtained from basic modeling, one having the least DOPE score of -8445.872 was chosen as the final structure (Fig. 5a). The modeled structure was superimposed upon 5TVI (template), which shows a low of RMSD 0.6 Å, suggesting that the suitable model is constructed under the range of ≤ 2 Å structural variation (Fig. 5b). Upon evaluating the predicted model by PROCHECK, it was found that 94.9% residues lie in the most favored region making the structure acceptable (Supplementary Figure S2). The -6.37 Z-score by the ProSA tool also indicated the model's accuracy within the scores of X-ray/NMR structures available in the PDB database (Supplementary Figure S3). The modeled caraway nsLTP1 structure can be accessed in PMDB (protein model database) [29] with ID PM0084405.

### Docking studies

**Fig. 6:**
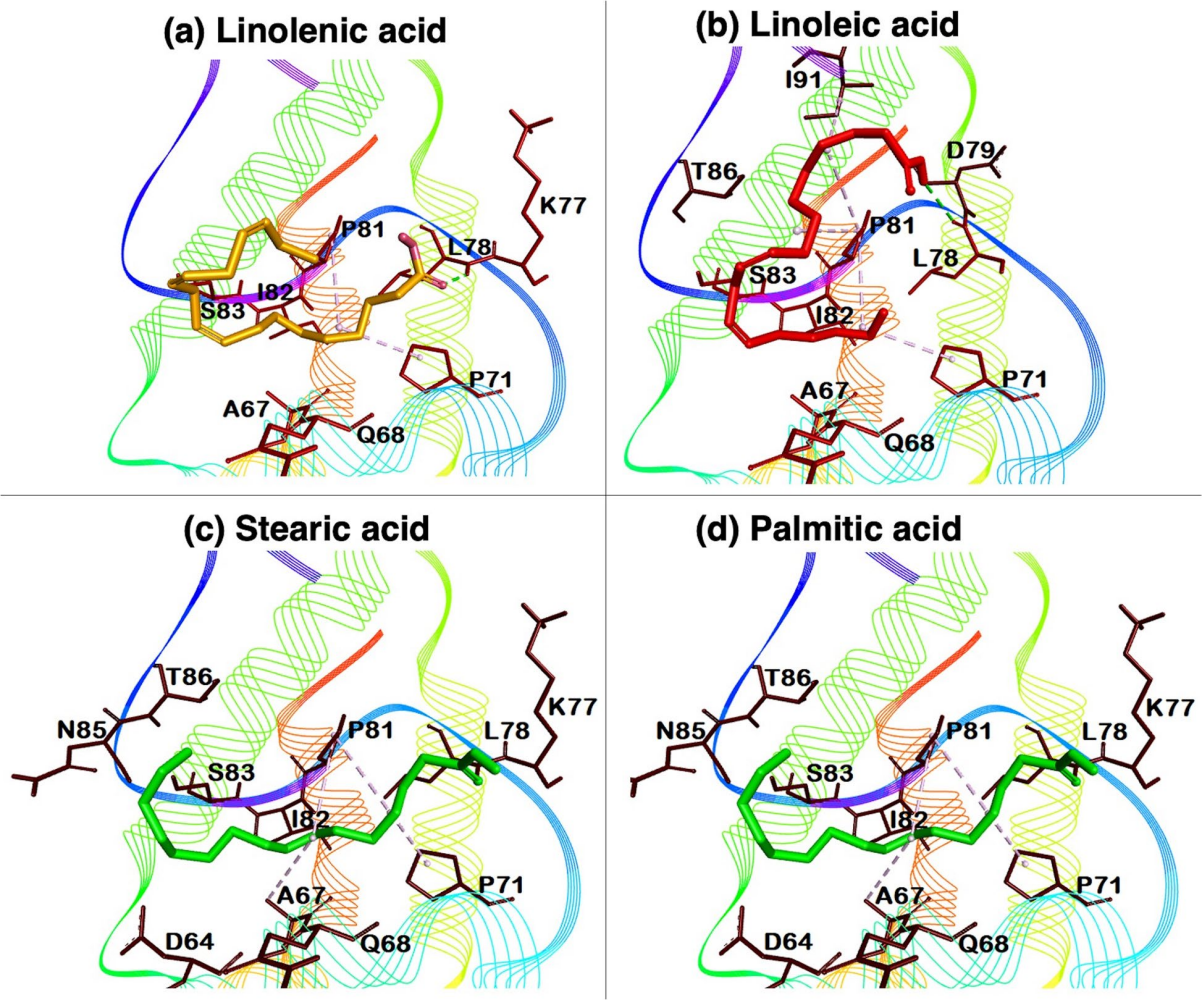
Binding conformations of four fatty acids with caraway nsLTP. **a** linolenic acid, (**b**) linoleic acid, (**c**) stearic acid, and (**d**) palmitic acid. The binding site of caraway nsLTP structure around 4Å is also labeled with amino acids

Figure 6 represents the docked complexes of linolenic, linoleic, stearic, and palmitic acid in the binding cavity of caraway nsLTP1. Linolenic acid with caraway protein showed a maximum binding score of -3.6 kcal/mol, whereas palmitic acid showed the least binding score of -3.0 kcal/mol. However, the other two fatty acid molecules, i.e., linoleic acid and stearic acid, exhibited -3.5 and-3.4 kcal/mol, respectively (Supplementary Figure S4). Further, the molecular interactions between caraway protein and ligands are tabulated in Supplementary table S2.

### Antiproliferative effect of nsLTP1

**Fig. 7:**
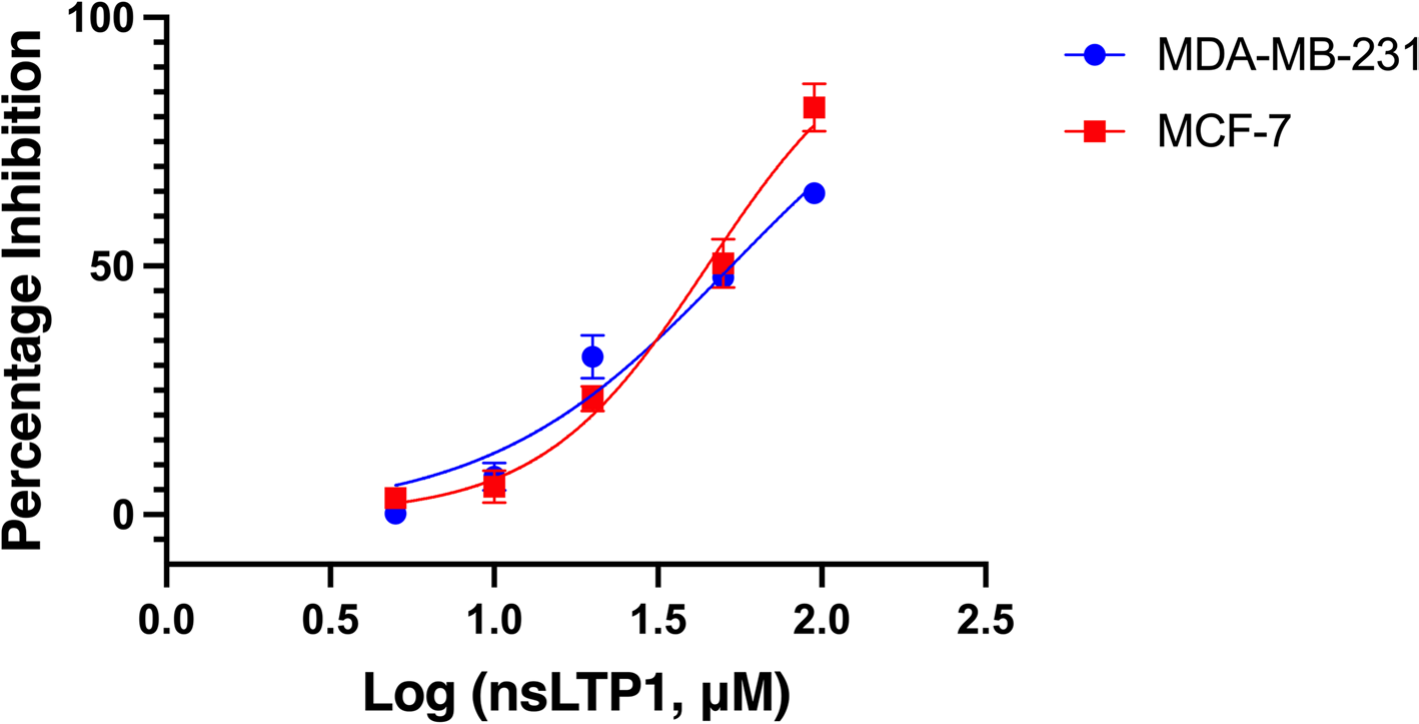
Dose-dependent cytotoxic activity of caraway nsLTP1 after 48 h treatment. Data from three independent experiments are presented with mean and standard deviation (SD). The IC_50_ was calculated using nonlinear regression analysis; For MDA-MB-231 cells, the value was 52.93 μM, while for MCF-7 cells, it was 44.76 μM

The antiproliferative activity of purified nsLTP1 from caraway seeds against MDA-MB-231 and MCF-7 cell lines was evaluated using the MTS assay. In a dose-dependent manner, the results showed inhibitory activity of nsLTP1 against MDA-MB231 and MCF-7 cells (Fig. 7). The IC_50_ was calculated using nonlinear regression analysis; For MDA-MB-231 cells, the value was 52.93 μM, while for MCF-7 cells, it was 44.76 μM. At the lowest concentration (5 μM), caraway nsLTP1 exhibited more inhibitory effect in MCF-7 cells than in MDA-MB-231 cells. Besides, at the highest concentration (95 μM), proliferation was inhibited by 65% and 82% for MDA-MB-231 and MCF-7, respectively.

### Antioxidant effect of nsLTP1

**Fig. 8:**
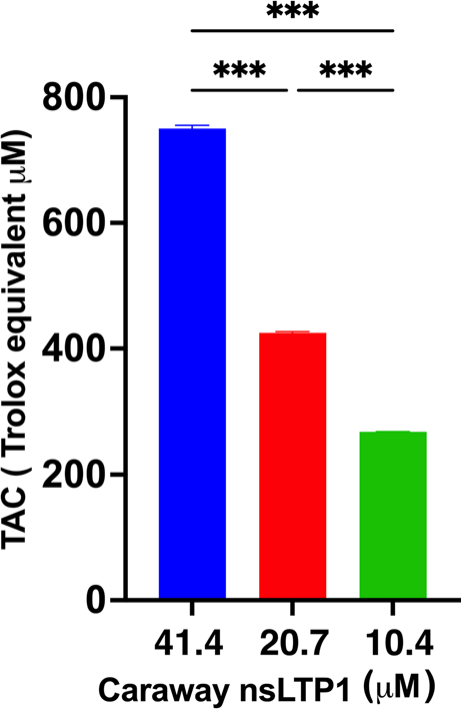
The total antioxidant capacity of caraway nsLTP1. For 41.4 μM caraway nsLTP1, the total antioxidant capacity was 750.4 ± 6.9 μM, and for 10.4 μM caraway nsLTP1, it was 268.2 μM ± 0.29 Trolox equivalent. Data is represented by Trolox equivalent with mean and standard deviation (SD) highlighted as *P < 0.05, **P < 0.01, ***P < 0.001

The antioxidant ability determination is inferred based on caraway nsLTP1 capability to reduce Cu2 + . The occurring Cu + primarily enhances the color of the solution by forming a colored complex in proportion to oxidation inhibition. Therefore, the sample displayed intense absorbance, confirming the protective effect of nsLTP1. The total antioxidant capacity (TAC) was dose-dependent, and the obtained value was 750.4 ± 6.9 μM Trolox equivalent at the highest concentration (41.4 μM). At the lowest concentration (10.4 μM), nsLTP1 showed a TAC value of 268.2 μM ± 0.29 μM Trolox equivalent (Fig. 8).

### CD analysis of nsLTP1 secondary structure

**Fig. 9:**
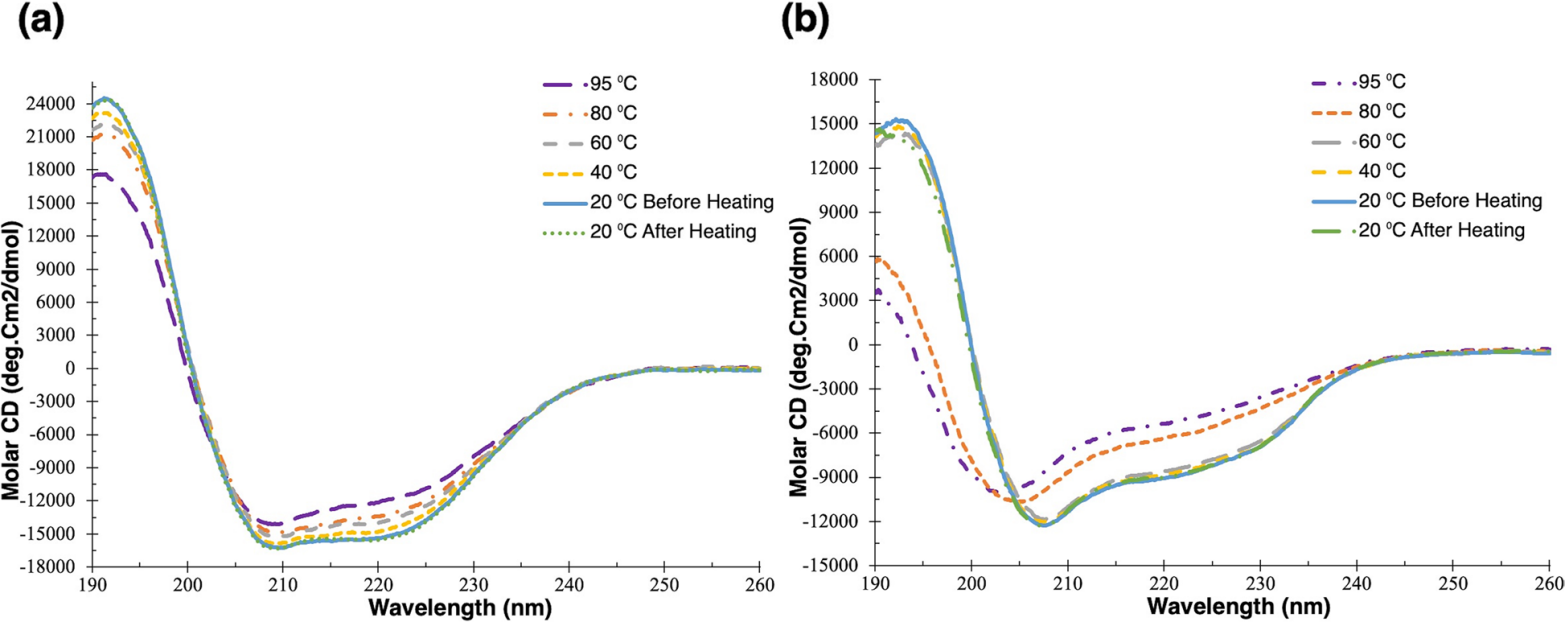
The thermal treatments effect on caraway nsLTP1 secondary structure. Caraway nsLTP1 (**A**) and Lysozyme (**B**) CD spectra were collected at 20, 40, 60, 80, and 95 °C and after cooling to 20 °C

The stability of caraway nsLTP1 under different temperatures, artificial gastric fluid, and artificial intestinal fluid was investigated using CD spectroscopy. First, the spectra were captured for nsLTP1 and lysozyme (positive control) at temperatures between 20 to 95 °C. Then, nsLTP1 and lysozyme were cooled to 20 °C to collect the data again (Fig. 9a). Lysozyme is reported to be stable in thermal treatments [30]. At high temperatures, the spectra changes of nsLTP1 were minimal, and the negative maxima remained stable. In addition, nsLTP1 completely returned to its original secondary structure when cooled to 20 °C. In contrast, lysozyme minimum shifts from 208 to 203 nm at high temperatures. Overall, lysozyme refolds almost completely once the temperature is reduced (Fig. 9b). Therefore, we can suggest that the nsLTP1 is resistant to thermal denaturation up to 95 °C. In addition, The data illustrates nsLTP1 superior thermal stability compared with lysozyme. Based on PCR's estimated secondary structure contents, α-helix is the predominant structure in nsLTP1 with a value of 34.2%, which increased slightly to 45.6% as the temperature rose to 95 ºC. The turn and other secondary structures had minor changes in higher temperatures, while the β-sheet secondary structures dropped to 7.5% from 27.8% at 95 °C (Table 1). nsLTP1 secondary structure stability was assessed in artificial gastric and intestinal fluids without proteolytic enzymes. Both fluids had some effects on the secondary structure, but there were minimal changes (Supplementary Figure S5). The spectra illustrated an increase and decrease in Molar CD intensity. The PCR analysis indicated a slightly lower content of β-sheet (AIF 2.4% and AGF 18.4%), almost unchanged turn (AIF 15.3% and AGF 13.0%), higher α-helix (AIF 46.4% and AGF 37.3%) and almost unchanged other (AIF 35.9% and AGF 31.3%) in artificial gastric fluid compared to nsLTP1 dissolved in ultrapure water (Supplementary table S3). Like thermal treatment results, percentages of α-helix increase and β-sheet decrease in AGF and AIF environments. The data suggest that nsLTP1 has a high tolerance to keep its secondary structure in the gastrointestinal tract without proteolytic enzymes.

**Table 1:** The temperature effect on the secondary structure of nsLTP1 and lysozyme estimated by PCR analysis of CD spectra

Temperature	α-helix		β-sheet		Turn		Other	
Sample	nsLTP1	Lysozyme	nsLTP1	Lysozyme	nsLTP1	Lysozyme	nsLTP1	Lysozyme
Before heating 20 ºC	34.2%	39.3%	27.8%	12.0%	8.5%	14.8%	29.5%	33.9%
40 ºC	37.6%	41.5%	23.3%	9.2%	9.4%	15.1%	29.7%	34.2%
60 ºC	42.2%	41.9%	17.3%	9.1%	10.3%	15.3%	30.2%	33.7%
80 ºC	43.5%	32.6%	15.0%	6.3%	10.9%	16.2%	30.6%	44.9%
95 ºC	45.6%	31.2%	7.5%	5.0%	12.7%	16.8%	34.2%	47.0%
After heating 20 ºC	31.3%	38.1%	30.2%	11.8%	8.2%	14.8%	30.3%	35.3%

## Discussion

Plants' lipid transfer proteins belong to a multigenic family performing multifunctional roles. Nevertheless, they exhibit particular common properties, i.e., small, basic nature, highly conserved conformational structures, and high stability [31]. Despite the efforts to define their biological roles in plants, and their studies are also driven by their active involvement in many aspects, e.g., lipid affinity, allergy, and drug delivery. Interestingly, they also displayed a variety of pharmacological effects, including antiproliferative, antimicrobial, and enzyme inhibition activities [5]. Therefore, here we isolate and characterize nsLTP1 from caraway seeds for the first time. Caraway proteomic studies are limited, and, to date, UniProt contains only UniProtKB/TrEMBL (unreviewed) sequences for caraway, and nsLTP1 is not listed. A novel antifungal peptide (AFP), Cc-AFP1, with a molecular weight of 3.7 kDa, was isolated from caraway [32]. It showed antifungal activity toward *Aspergillus* species with MIC ranging from 8–16 μg/ml. Although rare, proteins in caraway could trigger an allergy in individuals with spice allergies. These protein allergens were identified as analogs of Bet v 1 (17kD), profili 1 (15kD), and elongation factor 1 α (55 kDa) using immunoblotting and LC–MS/MS method [33]. From the Apiaceae family, caraway nsLTP1 is the seventh protein characterized, following fennel, ajwain, dill (*Anethum graveolens*), cumin (*Cuminum cyminum*), celery (*Apium graveolens*), and carrot. The molecular weight of caraway nsLTP1 is 9.6 kDa, similar to the rest of the Apiaceae family nsLTP1.

Caraway nsLTP1 is a single polypeptide chain consisting of 91 amino acid residues and possesses the structural conformation of nsLTP1. The primary structure, represented by the amino acid sequence, determines the protein tertiary structure. This three-dimensional structure defines its specific cellular role [34]. With the enlightening of the complete amino acid sequence of caraway nsLTP1, homology and computational structure studies were conducted for in-depth structure analysis. Homology modeling is suitable in silico approach for structure prediction when the template is greater than 30% identity. Blastp retrieved 16 sequences that produced significant alignments from the PDB database, which 5TVI (*S. melongena*) opted for as a suitable template with 48% identity and 67% similarity. This way, the best three dimensional structure of caraway nsLTP1 was constructed. The modeled structure exhibits basic features. There are four helices linked with each other by three loops positioned at H1 (4–19), H2 (26–38), H3 (43–58), and H4 (64–73), and the structure ends at the C-terminal tail from residues (75- 91). These four helices, in parallel, create the unique hydrophobic tunnel-like cavity, where it gains the ability to bind and transfer various ligands [35]. Hydrophobic residues are buried inside the cavity without interacting with each other constructing the ligand binding site. In addition, among the marked characteristics, the eight-cysteine motif (8CM) is conserved and implicated in forming the disulfide bridges. Like 5TVI, the stability of the modeled structure is retained by the formation of four disulfide bonds through 8 cysteine residues at positions CysI (4) – CysVI (51), CysII (14) – CysIII (28), CysIV (29) – CysVII (74), and Cys V (49)–CysVIII (88) [36].

In multiple sequence alignment, all the reported nsLTP sequences were aligned with the sequence of caraway nsLTP1. These nsLTPs show well-conserved sequences. However, a closer look indicated slight substitutions among all nsLTP sequences. Asp8 is retained in all nsLTPs except *M. esculenta* and *Dorcoceras hygrometricum* nsLTP. Pro13 and Pro71 are substituted by alanine in *Gossypium hirsutum* and *Nicotiana sylvestris* and Pro81 by valine in *Striga hermonthica* nsLTP. There is Phe17 and Ile76 in *M. esculenta* nsLTP, while the rest of the nsLTPs contains tyrosine and valine residues, respectively. Ala39 is replaced by serine in *C. Carvi*, *T. ammi,* and *D. carota* nsLTP. All *Solanum* nsLTPs follow the same substitution pattern, i.e., Leu/Met at 11, Thr/Ser at 41, and Asp/Asn at 79. Ile at 59 substitutes Tyrosine in *G. hirsutum* sequence. Replacement of Lys63 by arginine in *A. foeniculum* and Asp64 by glutamic acid in *G. hirsutum* were observed. Ile80 is replaced by Phe and Val in *G. hirsutum* and *N. sylvestris* sequences. Phe91 is found instead of Ile in *D. hygrometricum* sequence.

In the phylogenetic analysis, a well-defined set of nsLTP sequences was chosen at the time of tree construction. Protein sequences were used for analysis as they give a better idea to study evolution [37]. Hypothetical proteins were excluded as they are not defined to be translated into functional proteins [37]. Unrooted tree was used to determine the ancestral relationship among the taxonomy and to find the set of convergence or divergence by making clusters among a set of similar sequences [38]. Sequences having a bootstrap value above 60 were considered for tree construction because bootstrap defines the probability of a repetition of a sequence which is more likely to be present in a particular position of a tree [39]. Caraway belongs to Apiaceae (Umbelliferae) family, which includes 434 other genera. Caraway is more closely related to the ajwain, carrot, and fennel of this family. A sister relationship is also found between ajwain and caraway. However, the divergence is observed between caraway family members and the rest of the taxa present in the phylogenetic tree. The neighbor-joining method was used because of its simplicity and robustness as compared to other methods for tree construction. However, this method is considered good along with the use of p distance and is referred to as NJp method. The p distance of the phylogenetic tree was computed, and all the values were significantly found between 0 and 1, indicating the construction of a reliable tree [40].

Molecular docking studies were performed for detailed insight into interaction patterns. The binding pocket server identified Asp8, Leu11, Ser12, Cys14, Ile15, Leu18, Leu19, Val52, Ala55, Ala56, Ala57, Arg58, Tyr59, Pro60, Leu62, Lys63, Asp64, Ala67, Gln68, Leu70, Pro71, Leu78, Pro81, Ile82, Ser83, Arg84 and Thr86 amino acid residues responsible for the binding of fatty acids. The post-docking results showed that mainly Leu78 contributed to the formation of hydrogen bonds almost in all fatty acids. However, Thr86 is also found in the contribution of hydrogen bonds with stearic acid. Likewise, all four fatty acids exhibited the same hydrophobic interaction making an alkyl bond with Pro71 and Pro81. Gln68, Ile82, and Ser83 were commonly found in making van der Waal interactions as well as subtle changes of other amino acids in some cases. This interaction pattern is found primarily similar in the case of docking of fennel nsLTP1 [10]. Due to similar binding patterns, the fatty acids were ranked according to the binding affinity as linolenic acid > linoleic acid > stearic acid > palmitic acid.

There is an increased report of biological activity produced by proteins and peptides extracted from natural sources, including plants nsLTPs. Therefore, we assessed caraway nsLTP1 for cytotoxic and antioxidant bioactivity. Since fennel nsLTP1, the first nsLTP reported by our lab demonstrated cytotoxicity toward breast cancer MCF-7. It was followed by ajwain nsLTP1, the closest nsLTP1 to caraway nsLTP1, which showed selective cytotoxicity toward breast cancer MCF-7 and hardly affected non-tumorigenic breast MCF10A cells. Here, we widen the assessment by including triple negative highly aggressive MDA-MB-231 breast cancer cells. Caraway nsLTP1 exhibited a cytotoxic effect against MDA-MB-321 and MCF-7 cells with IC_50_ of 52.93 and 44.76 μM, respectively. Compared to previously reported nsLTP1 cytotoxic activity toward MCF-7 cells, fennel and ajwain showed IC_50_ of 6.98 μM and 8.21 μM, respectively, while mung bean (*Vigna radiata*) nsLTP1 showed no activity [8, 10, 41]. No previously characterized nsLTP1, aside from the present study, has been utilized to assess toxicity against MDA-MB-321, a metastatic and aggressive triple-negative breast cancer cell line. Although we report cytotoxic activity of nsLTP1 from caraway seeds towards them, but the mechanism of action needs to be identified. Nevertheless, when MCF-7 and ASPC-1 were treated with ajwain nsLTP1, apoptotic cascades of the Bcl-2 family displayed involvement in the mechanism of action [8, 10]. In addition, maize nsLTP1 showed sequence similarity to BID, a member of Bcl-2 proteins, and evoked the release of cytochrome C in a mitochondrial model [42, 43]. Anti-inflammatory effect by modulation of pro- and anti-inflammatory cytokines in vivo was reported from noni (*Morinda citrifolia*) seeds nsLTP1. The assumption of chemokines involvement could also be proposed to play a role in immunotherapy [44].

Efforts are in progress to explore natural antioxidants to lower the risks of oxidative stress linked to several diseases. The TAC of caraway nsLTP1 (41.4 μM) was evaluated and showed a capacity up to 750.4 μM Trolox equivalent. This finding is consistent with a previous nsLTP1 report. Barley (*Hordeum vulgare*) nsLTP1 exhibited free radical scavenging and a significant antioxidant capacity [45]. Interestingly, a study assessed the antioxidant effect of bread fortified with oil-free caraway seed (oil extraction by-product) and demonstrated its capability to produce antioxidant activity [46]. Such activity could be credited to caraway components other than the oil, in which nsLTP1 is among the content. The antioxidant activity of caraway seed extract and oil is significant and commonly recognized as the purpose of its other biological activities, including cytotoxicity [47].

With the advances in protein and peptide therapeutics, the assessment of protein stability is essential for drug development. Unfolding of proteins due to temperature and pH changes leads to protein denaturation and loss of biological activity. Thus, caraway nsLTP1 thermal, artificial gastric fluid, and artificial intestinal fluid stabilities were studied by CD spectroscopy. The secondary structure of α-helical model proteins have characteristic CD spectra [48]. We found that caraway nsLTP1 is a highly stable protein at 95 °C temperature and retained its original secondary structure after cooling to 20 °C. The thermostability of nsLTP1 could be possibly due to the presence of intramolecular disulfide bonds. The positive maxima at 191 nm and double minima at 220 nm and 209 nm were the unique signatures of the protein, as previously reported by Alshammari et al. (2022) for ajwain nsLTP1 [8]. In addition, our previous paper used collagen to show irreversible instability with thermal treatments by following the same method [8]. Nevertheless, a thermal denaturation occurred when hazelnut nsLTP1 in sodium phosphate at pH 7.5 was heated to 95 °C and lost its ability to refold after cooling [49]. Based on the PCR analysis at 20 °C, 34.2% of the protein is α-helix and 27.8% is β-sheet. These data are consistent with other studies. For example, the helices estimation for ajwain, amaranth, and celery nsLTP1 is reported to be 32.4%, 47%, and 40.3%, respectively [8, 50, 51]. This could be owed to the different characteristics of each nsLTP1 extracted from different sources. The data suggest that the caraway nsLTP1 is resistant to thermal denaturation and only showed minimal conformational changes.

To investigate caraway nsLTP1 stability in the digestive tract juices, it was dissolved in the artificial gastric and intestinal fluids. Some proteins are pH-dependent to carry out their functions and can be denatured in an undesirable pH environment. Pepsin and trypsin are aspartic and serine proteases. An optimal pH of around 2 for pepsin and 8 for trypsin permits them to operate their function while denatured at pH 7 and 4, respectively [52, 53]. Caraway nsLTP1 in artificial gastric pH 1.6 and intestinal pH 6.8 fluids almost conserved its secondary structure. Although the molar CD unit has increased in artificial intestinal fluid and decreased in artificial gastric fluid in contrast to the protein dissolved in ultrapure water, the overall structure was preserved, and the α-helical maximum at 191 and double minima at 209 and 220 were observed. At room temperature, hazelnut nsLTP1 dissolved in sodium phosphate also retained its structure at pH 7.5 and 2.5 [49]. There is a similar pattern in PCR analysis data in this study and the thermal one. As the temperature increases and pH changes, the α-helix increases, the β-sheet decreases, turn increases, and other structure increases. This indicates that the protein deals with changes in the environment in an organized and consistent manner to keep and conserve the secondary structure as much as possible. No proteolytic enzymes were used in either artificial gastric or intestinal fluids. In addition to the thermal stability explained before, nsLTP1 secondary structure shows minimal changes in the gastrointestinal tract fluids without proteolytic enzymes.

## Conclusion

In the present study, we isolated, purified, and characterized the amino acid sequence of a 9.6 kDa protein member of nsLTP1 from caraway (*C. carvi*) seeds. We also predicted the 3D structure modeling using eggplant (*S. melongena*) 5TVI as a template. Moreover, in silico studies for lipid affinity and evolutionary connections were conducted. NsLTPs appeal promising for drug discovery and development applications due to their revealed biological activities. Hence, this study also reports preliminary studies for the in vitro bioactivity of caraway nsLTP1. The protein displayed antiproliferative activity against MDA-MB-231 and MCF-7 human breast cancer cell lines. An in-depth molecular study is required to investigate the involvement of tumor suppressor proteins and signal transduction pathways in the process. Further confirmation of antiproliferative effects will require in vivo studies. Caraway seed also exhibited valued Trolox equivalent antioxidant capacity. Besides, caraway nsLTP1 showed high stability in temperatures ranging from 20–90 °C and stability in different pH conditions.

## Supplementary Information

**Additional file 1: Supplementary Table S1.** Template sequences obtained by BLASTp results against PDB database. **Supplementary Table S2.** The molecular interactions found between caraway nsLTP1 and linolenic, linoleic, stearic and palmitic acids ligands. **Supplementary Table S3.** Artificial fluids affect over caraway nsLTP1 secondary structure estimated by Principal Component Regression (PCR) analysis of CD spectra. **Supplementary Fig. S1.** The full-length electrophoretic profile by Tris/Tricine SDS-PAGE (10%) of caraway seeds proteins precipitates and gel filtration chromatography fractions. Lane M, standard molecular weight marker, Lane C, crude proteins, and Lane 1–8, eluted gel filtration fractions. **Supplementary Fig. S2.** Ramachandran Plot of modeled structure of caraway nsLTP1. Most favored region contains more than 90% residues predicting a good quality model. **Supplementary Fig. S3.** Validation of modeled caraway nsLTP1 structure through ProSA server. Z-score is -6.37 within the range of scores of experimentally determined structures. **Supplementary Fig. S4.** 2D interactions drawn from Discovery Studio of four fatty acids complexed with caraway nsLTP1 (a) linolenic acid (b) linoleic acid (c) stearic acid (d) palmitic acid are making hydrogen bond, van der waal interaction and alkyl (hydrophobic) interaction. **Supplementary Fig. S5.** Stability of caraway nsLTP1 under different pH environments. Caraway nsLTP1 in ultrapure water, artificial gastric fluid pH 1.6, and artificial intestinal fluid pH 6.8 CD spectra were collected at 37 °C.

## Data Availability

The datasets generated or analyzed during the current study are part of the T.A. ongoing doctoral dissertation but are available from the corresponding author upon reasonable request.

## Abbreviations

nsLTP: Nonspecific lipid transfer protein
CD: Circular dichroism
TAC: Total antioxidant capacity
IC_50_: Half-maximal inhibitory concentration
PBS: Phosphate buffered saline
SDS-PAGE: Sodium dodecyl sulfate–polyacrylamide gel electrophoresis
MALDI: Matrix-assisted laser desorption/ionization
RP-HPLC: Reverse-phase high pressure liquid chromatography
βME: 2-Mercaptoethanol
TPCK-treated: L-(tosylamido-2-phenyl) ethyl chloromethyl ketone treated
CNBr: Cyanogen Bromide
PTH: Phenylthiohydantoin
PVDF: Polyvinylidene difluoride
DMSO: Dimethyl sulfoxide
E-value: Expect value
PCR: Principal component regression
MTS: 3-(4,5-Dimethylthiazol-2-yl)-5-(3-carboxymethoxyphenyl)-2-(4-sulfophenyl)-2H-tetrazolium
Bcl-2: B-cell lymphocyte 2
