# Supplementary material for: The structural characterization and bioactivity assessment of nonspecific lipid transfer protein 1 (nsLTP1) from caraway (Carum carvi) seeds
Source: BMC Complement Med Ther. 2023 Jul 20;23:254. doi: 10.1186/s12906-023-04083-9 (PMC10357877; doi:10.1186/s12906-023-04083-9)

The structural characterization and bioactivity assessment of nonspecific lipid transfer protein 1 (nsLTP1) from caraway (*Carum carvi*) seeds

Taibah Aldakhil[1,2], Saud O. Alshammari[1,3], Bushra Siraj[4], Bishoy El-Aarag[1,5], Shamshad Zarina[4], David Salehi[1], Aftab Ahmed[1, *]

[1] Biomedical and Pharmaceutical Sciences, Chapman University School of Pharmacy, Irvine, CA 92618, USA

[2] Department of Pharmaceutical Chemistry, College of Pharmacy, Prince Sattam Bin Abdulaziz University, Al-Kharj 16278, Saudi Arabia

[3] Department of Plant Chemistry and Natural Products, Faculty of Pharmacy, Northern Border University, Arar 91431, Saudi Arabia

4. Dr. Zafar H. Zaidi Center for Proteomics, University of Karachi, Karachi, Pakistan

5. Biochemistry Division, Chemistry Department, Faculty of Science, Menoufia University, Shebin El-Koom 32512, Egypt

*Corresponding author

Aftab Ahmed
Chapman University School of Pharmacy
9401 Jeronimo Road
Irvine, CA 92618
USA
Tel: (714) 516-5465
Fax: (714) 516-5481
aahmed@chapman.edu

Supplementary Table S1: Template sequences obtained by BLASTp results against PDB database.

S. No.	Organism Name	Total score	Query coverage	E-value	Percent Identity	Positives	PDB ID
1.	*Solanum melongena*	100	97%	6E-29	47.78	67	5TVI_V
2.	*Actinidia chinensis var. chinensis*	100	97%	7E-29	50.00	68	7KSB_A
3.	*Prunus persica*	76.3	97%	3E-19	40.00	58	2ALG_A
4.	*Lens culinaris*	72.8	97%	6E-18	42.22	55	5LQV_A
5.	*Lens culinaris*	72.8	97%	6E-18	42.22	55	2MAL_A
6.	*Corylus avellana*	70.5	98%	5E-17	38.46	54	4XUW_A
7.	*Oryza sativa*	70.1	97%	6E-17	37.78	56	1BV2_A
8.	*Zea mays*	70.1	97%	8E-17	39.56	58	1AFH_A
9.	*Triticum aestivum*	68.9	97%	1E-16	35.56	58	1BWO_A
10.	*Artemisia vulgaris*	65.9	97%	3E-15	36.67	53	6FRR_A
11.	*Vigna radiata var. radiate*	65.5	97%	4E-15	38.89	53	1SIY_A
12.	*Pisum sativum*	63.5	97%	3E-14	39.13	56	2N81_A
13.	*Hordeum vulgare*	61.2	97%	2E-13	31.11	55	1BE2_A
14.	*Punica granatum*	59.7	97%	8E-13	34.07	54	7KSC_A
15.	*Nicotiana tabacum*	58.9	97%	1E-12	36.67	54	1T12_A
16.	*Anethum graveolens*	57.8	97%	4E-12	36.56	52	2N2Z_A

Supplementary Table S2: The molecular interactions found between caraway nsLTP1 and linolenic, linoleic, stearic and palmitic acids ligands.

COMPOUNDS	VINA SCORES	HYDROGEN BOND (Å)	HYDROPHOBIC INTERACTIONS	VAN DER WAALS INTERACTIONS
Linolenic acid	-3.6 Kcal/mol	Leu78 (2.1 Å)	Pro71 (Alkyl)<br>Pro81 (Alkyl)	Ala67, Gln68, Lys77, Ile82 and Ser83
Linoleic acid	-3.5 Kcal/mol	Leu78 (2.8 Å)	Pro71 (Alkyl)<br>Pro81 (Alkyl)<br>Ile91 (Alkyl)	Ala67, Gln68, Asp79, Ile82, Ser83 and Thr86
Stearic acid	-3.4 Kcal/mol	Thr86 (1.9 Å)	Ala67 (Alkyl)<br>Pro71 (Alkyl)<br>Pro81 (Alkyl)	Asp64, Gln68, Leu78, Ile82, Ser83 and Asn85
Palmitic acid	-3.0 Kcal/mol	Leu78 (2.0 Å)	Ala67 (Alkyl)<br>Pro71 (Alkyl)<br>Pro81 (Alkyl)	Asp64, Gln68, Lys77, Ile82, Ser83, Asn85 and Thr86

Supplementary Table S3: Artificial fluids affect over caraway nsLTP1 secondary structure estimated by Principal Component Regression (PCR) analysis of CD spectra.

Temperature	α-helix	β-sheet	Turn	Other
Ultrapure Water	32.4%	21.9%	11.2%	34.5%
AIF*	46.4%	2.4%	15.3%	35.9%
AGF*	37.3%	18.4%	13.0%	31.3%

*Artificial Gastric (AGF) and Intestinal (AIF) Fluids.

Supplementary Fig. S1. The full-length electrophoretic profile by Tris/Tricine SDS-PAGE (10%) of caraway seeds proteins precipitates and gel filtration chromatography fractions. Lane M, standard molecular weight marker, Lane C, crude proteins, and Lane 1–8, eluted gel filtration fractions.

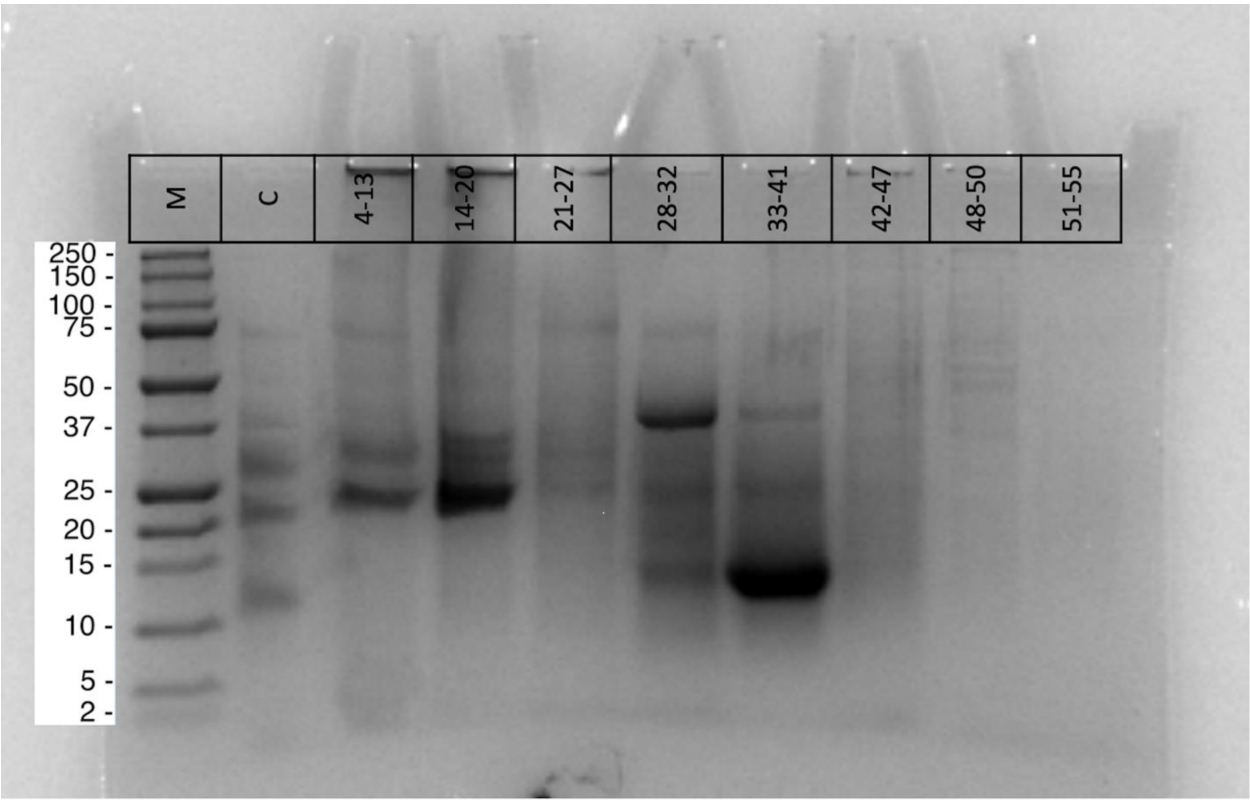

Supplementary Fig. S2. Ramachandran Plot of modeled structure of caraway nsLTP1. Most favored region contains more than 90% residues predicting a good quality model.

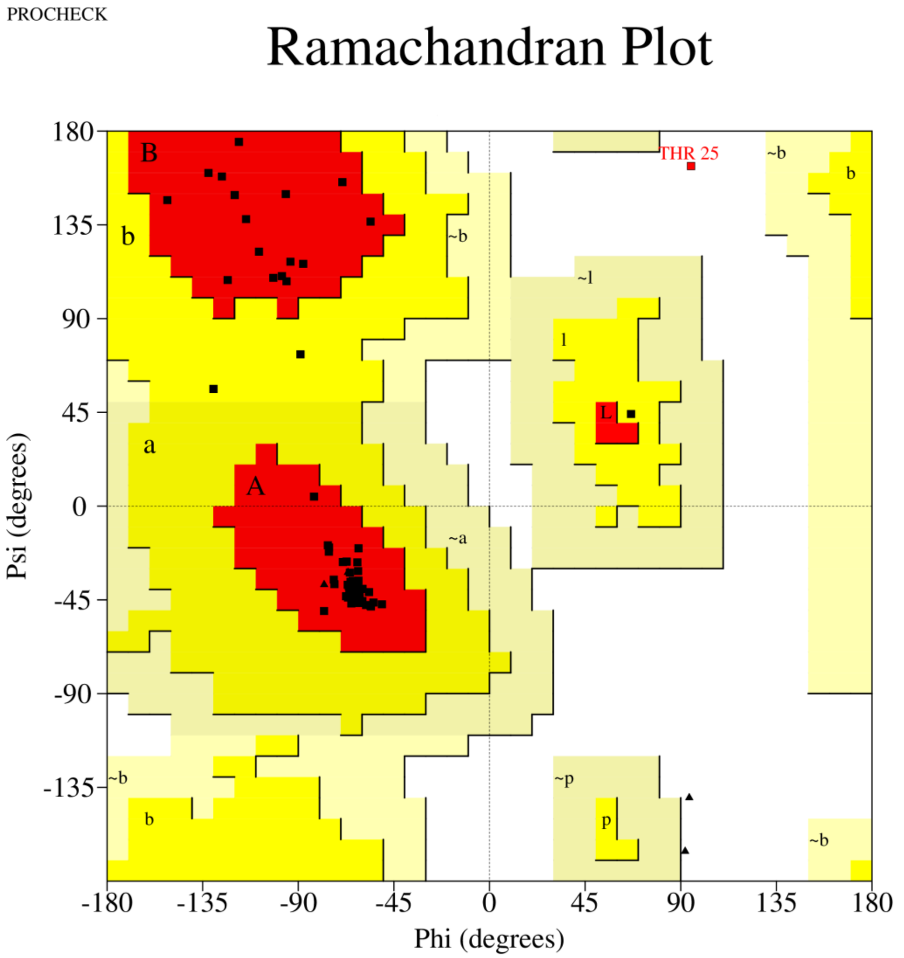

Plot statistics

Residues in most favoured regions [A,B,L]	74	94.9%
Residues in additional allowed regions [a,b,l,p]	3	3.8%
Residues in generously allowed regions [~a,~b,~l,~p]	0	0.0%
Residues in disallowed regions	1	1.3%
	----	------
Number of non-glycine and non-proline residues	78	100.0%
Number of end-residues (excl. Gly and Pro)	2	
Number of glycine residues (shown as triangles)	5	
Number of proline residues	6	

Total number of residues	91	

Based on an analysis of 118 structures of resolution of at least 2.0 Angstroms and R-factor no greater than 20%, a good quality model would be expected to have over 90% in the most favoured regions.

Supplementary Fig. S3. Validation of modeled caraway nsLTP1 structure through ProSA server. Z-score is -6.37 within the range of scores of experimentally determined structures.

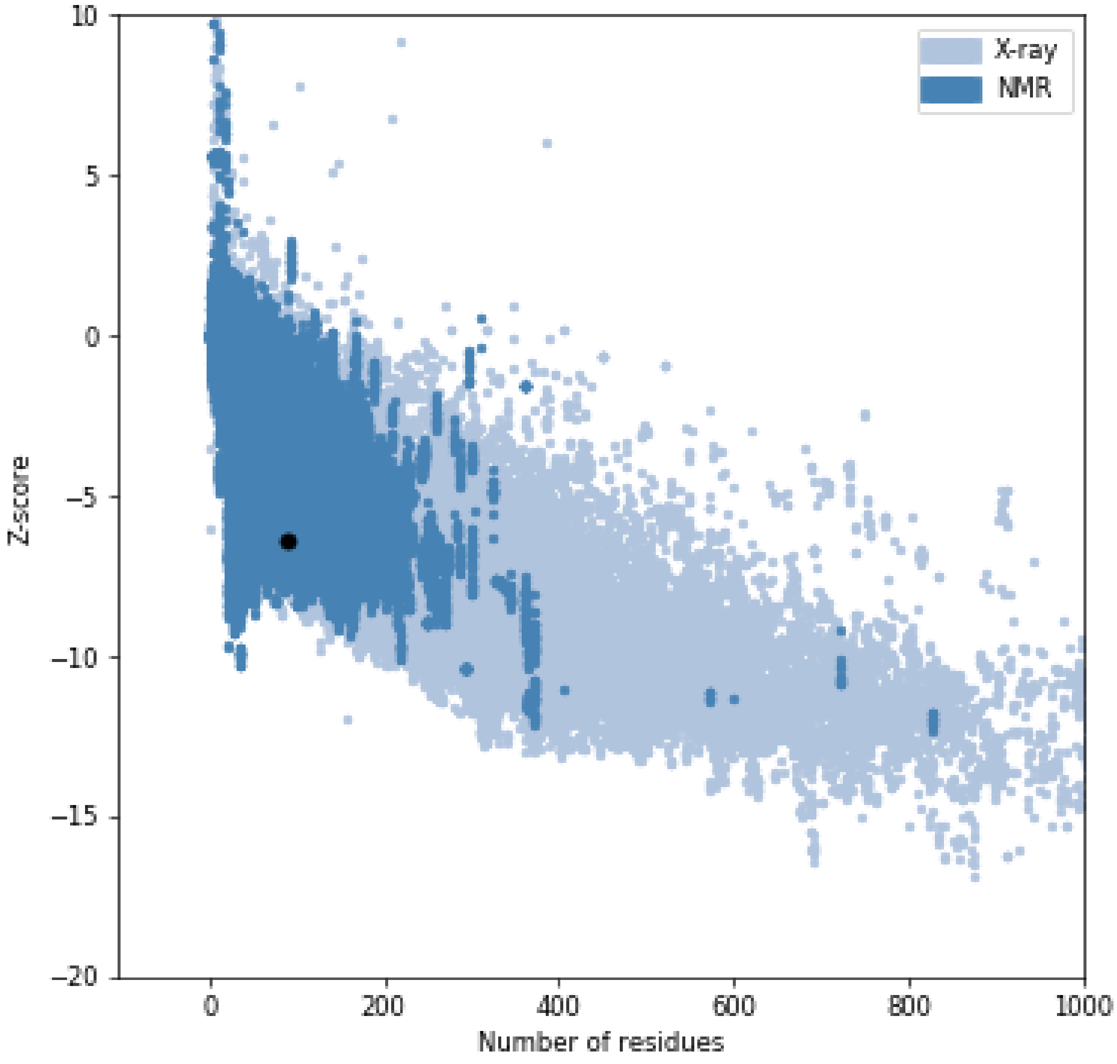

Supplementary Fig. S4. 2D interactions drawn from Discovery Studio of four fatty acids complexed with caraway nsLTP1 (a) linolenic acid (b) linoleic acid (c) stearic acid (d) palmitic acid are making hydrogen bond, van der waal interaction and alkyl (hydrophobic) interaction.

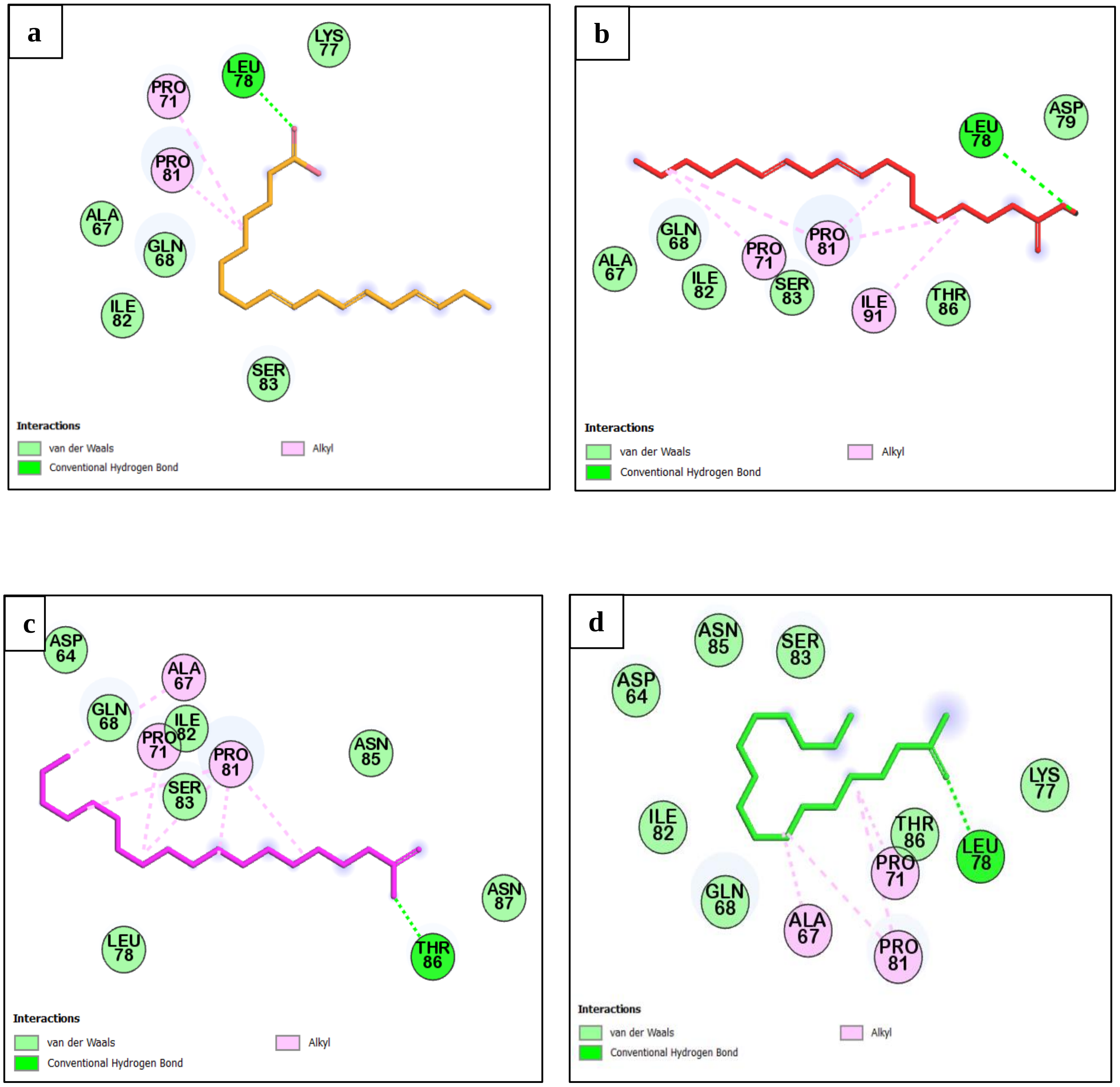

Supplementary Fig. S5. Stability of caraway nsLTP1 under different pH environments. Caraway nsLTP1 in ultrapure water, artificial gastric fluid pH 1.6, and artificial intestinal fluid pH 6.8 CD spectra were collected at 37 °C.

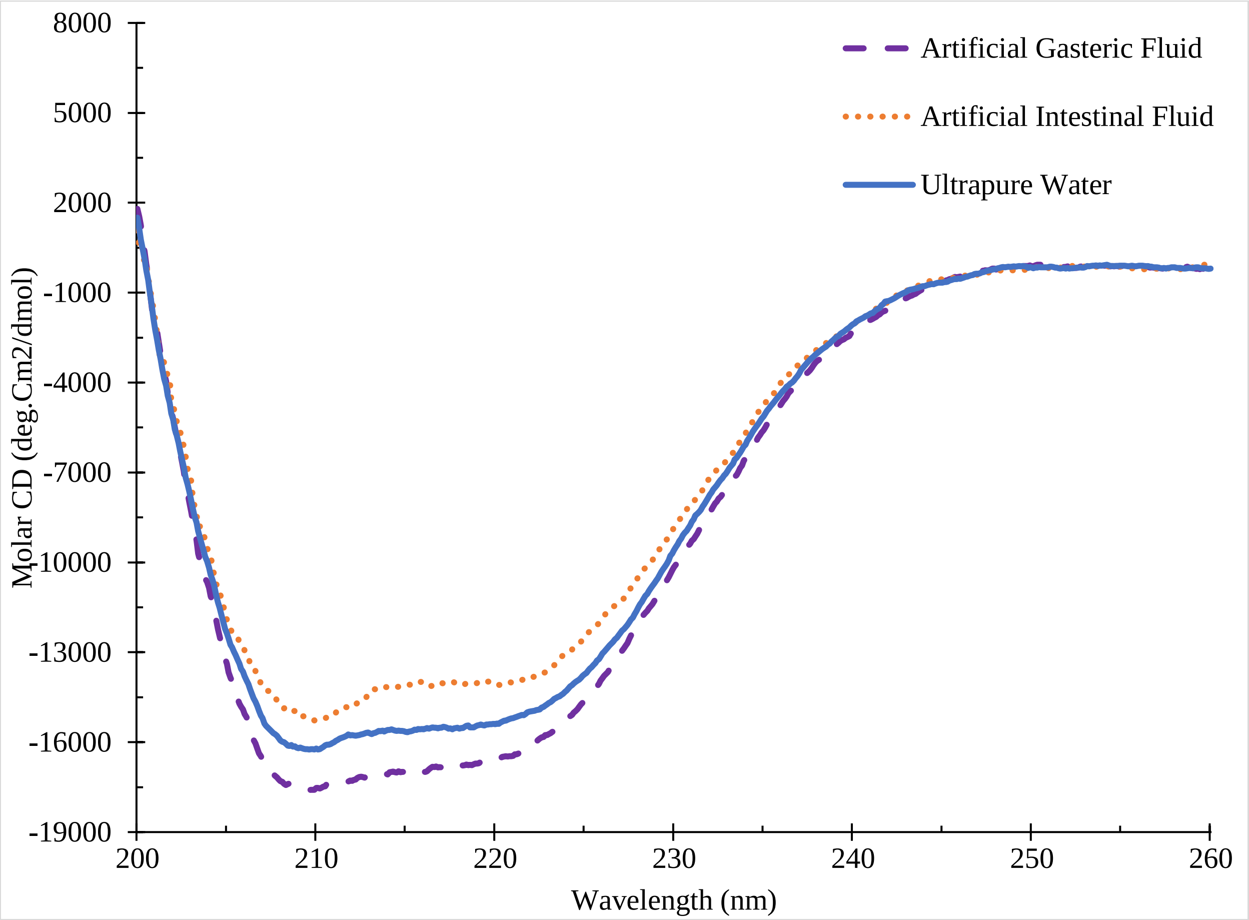

Supplement: Supplementary file 1 — Additional file 1: Supplementary Table S1. Template sequences obtained by BLASTp results against PDB database. Supplementary Table S2. The molecular interactions found between caraway nsLTP1 and linolenic, linoleic, stearic and palmitic acids ligands. Supplementary Table S3. Artificial fluids affect over caraway nsLTP1 secondary structure estimated by Principal Component Regression (PCR) analysis of CD spectra. Supplementary Fig. S1. The full-length electrophoretic profile by Tris/Tricine SDS-PAGE (10%) of caraway seeds proteins precipitates and gel filtration chromatography fractions. Lane M, standard molecular weight marker, Lane C, crude proteins, and Lane 1–8, eluted gel filtration fractions. Supplementary Fig. S2. Ramachandran Plot of modeled structure of caraway nsLTP1. Most favored region contains more than 90% residues predicting a good quality model. Supplementary Fig. S3. Validation of modeled caraway nsLTP1 structure through ProSA server. Z-score is -6.37 within the range of scores of experimentally determined structures. Supplementary Fig. S4. 2D interactions drawn from Discovery Studio of four fatty acids complexed with caraway nsLTP1 (a) linolenic acid (b) linoleic acid (c) stearic acid (d) palmitic acid are making hydrogen bond, van der waal interaction and alkyl (hydrophobic) interaction. Supplementary Fig. S5. Stability of caraway nsLTP1 under different pH environments. Caraway nsLTP1 in ultrapure water, artificial gastric fluid pH 1.6, and artificial intestinal fluid pH 6.8 CD spectra were collected at 37 °C. [file 12906_2023_4083_MOESM1_ESM.pdf]
